# Evaluation of multi-target deep neural network models for compound potency prediction under increasingly challenging test conditions

**DOI:** 10.1007/s10822-021-00376-8

**Published:** 2021-02-17

**Authors:** Raquel Rodríguez-Pérez, Jürgen Bajorath

**Affiliations:** 1 Department of Life Science Informatics, B-IT, LIMES Program Unit Chemical Biology and Medicinal Chemistry, Rheinische Friedrich-Wilhelms-Universität, Friedrich-Hirzebruch-Allee 6, 53115 Bonn, Germany; 2 Present Address: Novartis Institutes for Biomedical Research, Novartis Campus, 4002 Basel, Switzerland

**Keywords:** Machine learning, Structure–activity relationships, Multi-target learning, Deep neural networks, Model validation

## Abstract

Machine learning (ML) enables modeling of quantitative structure–activity relationships (QSAR) and compound potency predictions. Recently, multi-target QSAR models have been gaining increasing attention. Simultaneous compound potency predictions for multiple targets can be carried out using ensembles of independently derived target-based QSAR models or in a more integrated and advanced manner using multi-target deep neural networks (MT-DNNs). Herein, single-target and multi-target ML models were systematically compared on a large scale in compound potency value predictions for 270 human targets. By design, this large-magnitude evaluation has been a special feature of our study. To these ends, MT-DNN, single-target DNN (ST-DNN), support vector regression (SVR), and random forest regression (RFR) models were implemented. Different test systems were defined to benchmark these ML methods under conditions of varying complexity. Source compounds were divided into training and test sets in a compound- or analog series-based manner taking target information into account. Data partitioning approaches used for model training and evaluation were shown to influence the relative performance of ML methods, especially for the most challenging compound data sets. For example, the performance of MT-DNNs with per-target models yielded superior performance compared to single-target models. For a test compound or its analogs, the availability of potency measurements for multiple targets affected model performance, revealing the influence of ML synergies.

## Introduction

Machine learning (ML) models are used to relate the chemical structure of compounds to their biological activity and derive qualitative or quantitative structure–activity relationship (Q)SAR models [1–3]. Supervised modeling of such relationships and compound activity or potency prediction is facilitated via classification (i.e., prediction of active/inactive states) or regression (i.e., potency value prediction) [4, 5]. ML methods have become important components of (Q)SAR analysis and especially deep learning (DL) approaches have recently attracted increasing interest in the QSAR field and beyond [6–8].

Deep neural networks (DNNs) have become a method of choice for many investigations, although significant advantages compared to other ML methods are not always evident, especially in compound activity/potency predictions. Despite their high complexity, low interpretability, and the large number of hyper-parameters that need to be optimized, DNNs have been employed to model a variety of data, predict different assay outcomes or various compound properties, and yielded promising results in many instances [7, 9–12]. Some studies have indicated potential benefits of DL in medicinal chemistry and drug design [6, 13, 14]. DNN models have mostly advanced new applications that were not operable with conventional ML methods. However, superior performance of DNNs in ML-based QSAR models has not been consistently observed in applications using data sets of different origins and composition [7, 12].

In this context, one of the most promising applications of DNNs is multi-task or multi-target (MT) learning [15, 16]. DNNs can be configured for MT learning, resulting in MT-DNNs, which aim to simultaneously predict compound activity or potency against multiple biological targets [16, 17]. Different studies have shown that MT-DNN modeling can further increase the predictive performance of other ML methods depending on data characteristics and volumes, hyper-parameter optimization, target correlation, and/or the fraction of missing data missing data in a multi-target matrix [7, 12, 17–19]. Compared to single-target modeling, the performance of MT-DNNs principally benefits from the presence of correlated predictions tasks. However, given the high level of complexity of MT-DNNs, there is considerable interest in increasing the understanding how these models should best be calibrated and optimized and how they should be properly evaluated in practical applications. Of note, in addition to DNNs, other ML architectures and models are also amenable to and benefit from MT configurations [16]. This makes it interesting to ascertain whether apparent benefits from MT-DNNs for multi-target activity predictions result from the algorithmic DL architecture and/or the MT configuration. Therefore, we aim to better understand under which conditions MT learning offers advantages over other prediction strategies and how varying conditions might influence the relative performance of different methods.

In this work, we have investigated how single-target (ST) and MT ML approaches compare if potency predictions are attempted under varying conditions on a large scale by investigating as many targets and compound classes as possible. Therefore, ST and MT models have been implemented and evaluated using different data division (splitting) strategies, which have been found to affect model performance. Compared to other approaches, MT-DNN models provided incremental advantages for predicting the most challenging activity classes.

## Methods and materials

### Compound activity data

Activity classes with high confidence activity data were extracted from ChEMBL version 24 [20]. In each case, a maximal confidence score of nine was required for direct binding assays with single human targets and equilibrium constants (K_i_ values) were exclusively selected as potency measurements. For compounds having multiple K_i_ values, the geometric mean was calculated if all potency values fell within the same order of magnitude and the mean pK_i_ value was larger than five. If not, the compounds were omitted. Thus, borderline active compounds were excluded from modeling. The resulting data set was composed of 70,491 compounds with activity against 847 human proteins and a total of 116,881 pK_i_ annotations. From the data set, analog series were systematically extracted using an algorithm [21] based upon the matched molecular pair formalism [22]. Activity classes containing less than 50 compounds and/or less than two series of analogs were excluded from the analysis. Accordingly, the final data set used for modeling comprised 66,977 compounds with activity against 270 targets. A total of 110,358 pK_i_ annotations were available, corresponding to 0.61% density of the corresponding compound-target matrix.

### Molecular representation

Compounds were represented using circular topological fingerprints. An in-house version of the extended-connectivity fingerprint with bond diameter 4 (ECFP4) [23] based upon the *OpenEye OEChem toolkit* [24] was used to generate atom environments for compounds and encode them using a hashing function. Modulo mapping was applied to convert variably-sized compound-based ECFP4 feature sets into a constantly-sized folded version consisting of 1024 bits.

### Calculation protocol

ML regression models were built to predict compound potency from chemical structure. Compounds were defined by the feature vector $\mathbf{x}$ or ECFP4 and one or more numerical potency values $y$ (one per target). Since the equilibrium constant values are log-normally distributed, compound labels $\mathbf{y}$ consisted of pK_i_ values [pK_i_ = − log_10_(K_i_)]. Given the negative sign, higher pK_i_ values indicate increasing compound potency. For different test systems and tasks, ST-DNN and MT-DNN models were built based upon corresponding training sets and applied to predict different test sets in three independent trials. Model hyper-parameters were optimized as described below. ST models were also built using random forest regression (RFR) and support vector regression (SVR). The calculation protocol was implemented in R and Python.

### Performance evaluation

Different measures were used to assess model performance including mean absolute error (MAE), median absolute error (MedAE), mean squared error (MSE), and correlation coefficient (r). The most frequently calculated MAE and MSE measures are defined in Eqs. (1) and (2), respectively:

1 $$MAE(y,\hat{y}) = \frac{1}{n}\sum\limits_{i = 1}^{n} {|y_{i} - \hat{y}_{i} |^{{}} }$$

2 $$MSE(y,\hat{y}) = \frac{1}{n}\sum\limits_{i = 1}^{n} {(y_{i} - \hat{y}_{i} )^{2} }$$

where *n* is the number of compounds, $y$ is the experimental potency (pK_i_) value and $\widehat{y}$ the predicted value.

### Machine learning algorithms

#### Support vector regression

SVR derives a regression function $f({\mathbf{x}}) = \left\langle {{\mathbf{w}},{\mathbf{x}}} \right\rangle + b$ where the weights (**w**) and bias (b) are obtained through an optimization task. The algorithm only tolerates a given amount of deviations from observed and predicted values in the training data (*ε*) by penalizing larger errors [25, 26]. Thus, so-called *ε*-insensitive tube SVR considers a tube of radius *ε* around the target values such that only the data points outside the tube are penalized. Slack variables can be introduced during optimization to relax the ε-tube conditions. The hyper-parameter C or cost factor can be considered as a trade-off to balance error minimization and model complexity, which might lead to overfitting. This regularization term penalizes large slack variables or deviations from the *ε*-tube. The optimization problem is solved in its Lagrange dual formulation, where ${\alpha }_{i}$ and ${{\alpha }_{i}}^{*}$ multipliers are introduced for each data point, yielding the following prediction function as a result $f\left(\mathbf{x}\right)= {\sum }_{i}\left({\alpha }_{i}-{{\alpha }_{i}}^{*}\right) \langle {\mathbf{x}}_{\mathbf{i}},\mathbf{x}\rangle$. This function only uses support vectors (training compounds outside the *ε*-tube) to facilitate a prediction. SVR models are rendered non-linear through the “kernel trick”, which replaces the scalar product by a kernel function to map feature vectors into higher-dimensional space. Herein, the non-linear Tanimoto kernel was used.

#### Random forest regression

RFR consists of an ensemble of decision trees in order to reduce the variance of individual trees [27]. During training, bootstrap aggregation yields decision trees with different (replaced) compound subsets. In the construction of each tree, best data splits are determined through node-based splitting where only a random subset of features is considered, which further reduces correlation across trees. MSE was used to assess the quality of a split. RFR predicts final potency values as the mean prediction across all decision trees.

#### Feedforward deep neural networks

A feedforward DNN is constituted by basis functions (neurons), which are organized in sequential layers. The prototypic DNN architecture includes an input layer, at least two hidden layers, and an output layer. The input layer comprises as many neurons as features, in this case 1024, while the model assigns one output neuron to each target. Neurons receive input values $\mathbf{x}$ from the previous layer, which are linearly combined with weights (**w**) and biases (b). Then, an activation function (h) is applied to obtain the neuron’s output. Thus, the output is given by the equation ${y}_{j}=h({\sum }_{i}{{\mathbf{w}}_{ji}}^{n} {\mathbf{x}}_{i}+ {{\mathrm{b}}_{j}}^{n})$, where *n* indicates the layer number [28, 29]. DNN models are trained by determining the weights for each neuron leading to the desired output. In this case, experimental and predicted pK_i_ values are compared using a loss function. Backpropagation calculates the gradient of the error function with respect to the weights, which is minimized using gradient descent. Thus, weights are iteratively updated in the direction of the negative gradient to minimize the error.

#### Calculations

SVR and RFR calculations were carried out using *scikit-learn* [30], whereas DNN models were implemented with *TensorFlow* [31] and *Keras* [32].

### Missing potency annotations

Potency values were sparsely distributed across the compound-target interaction matrix (density 0.61%; see above). ST models were trained with a single potency value per compound. Therefore, missing potency annotations did not influence ST model building. By contrast, in the case of MT-DNN, all tasks are modeled simultaneously. Therefore, each compound label was assigned a vector of length 270, i.e. assigning one potency value per target, and a custom loss function masking missing labels was created. With this function, the model only uses existing data points when computing the training loss. Thus, only available compound-target potency annotations were used for model training. In target-based data splitting, there might be compounds with activity annotations both in the training and test sets (for different targets). In this case, compound potency values belonging to the test set were also masked during training.

### Hyper-parameter optimization

For each model and independent trial, selected model hyper-parameters were optimized through two-fold cross-validation using training data and a grid search to minimize the MSE. In RFR, three candidate values were considered for the minimum number of samples required to split a node (2, 8, 16) and the minimum number of samples in a leaf node (1, 5, 10). In the search for the best data split, possible values for the maximum number of features were the square root or logarithm (base 2). Finally, the number of trees in the ensemble was set to 500. The candidate values most frequently selected as the best solution for RFR models during the grid search included log_2_ (maximum number of features) and 3 (minimum samples leaf).

SVR models used a customized Tanimoto kernel, and only the cost value C was optimized during cross-validation (0.001, 0.01, 0.1, 1, 10, 100). Optimized SVR models preferentially employed a cost value of 0.01.

DNN models were trained with different learning rates (0.01, 0.001, 0.0001). Grid search was also applied to drop-out rates (0.1, 0.25, 0.4) and batch size (64, 128). Alternative network architectures were tested including different numbers of hidden layers (2, 3, 4) with rectified linear unit (ReLU) activation and neurons (100, 200, 1000, 2000). Networks were trained with or without batch normalization and the Adam optimizer was used. The final models were trained for early termination and a maximum of 200 epochs. For ST-DNNs, combinations of preferred hyper-parameter values were frequently observed leading low MSE. For example, learning rates of 0.001 or 0.0001 were preferred over 0.01. Increasing the dropout rate had an effect for some hyper-parameter combinations, leading to higher errors. Furthermore, a dropout rate of 0.1 was generally preferred. The most frequently selected hyper-parameters for MT-DNNs included two hidden layers with 2000 and 1000 neurons, respectively, without batch normalization, a batch size of 128, drop-out rate of 0.1, and learning rate of 0.0001.

## Results and discussion

Multi-target (MT) and single-target (ST) ML models were built to predict compound potency values for activity classes covering 270 biological targets. Initially, a large set of compound activity data was obtained from ChEMBL, which only included assays measuring direct binding to human targets with available K_i_ values. Following data curation, the resulting set used for modeling comprised 66,977 compounds, and 100,358 compound-target potency annotations (corresponding to 0.61% density of the corresponding compound-target matrix). Next, regression models were built to systematically predict the logarithmic potency (pK_i_) values for individual targets or multiple targets simultaneously.

### Data division strategies

**Fig. 1:**
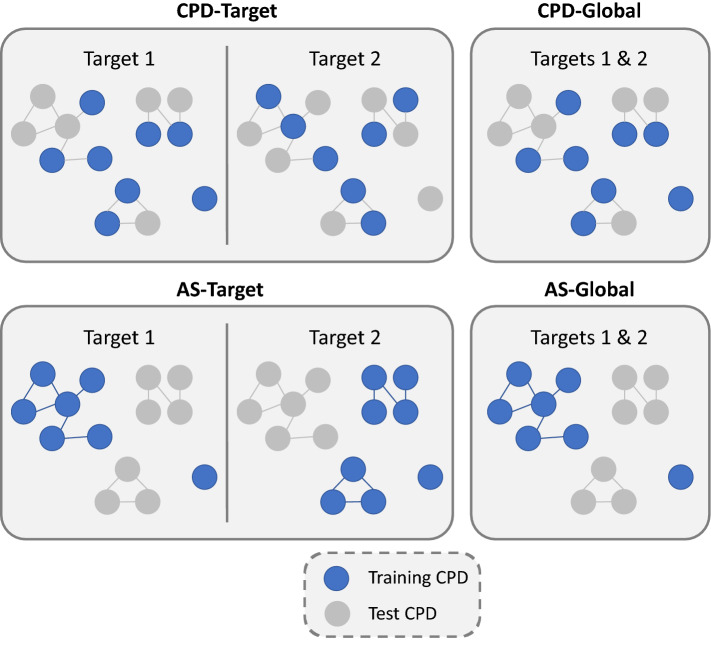
Data division strategies. The four data splitting schemes (CPD-Target, CPD-Global, AS-Target, and AS-Global) are schematically illustrated. Nodes represent compounds that are connected by an edge node if they form an MMP. Disjoint clusters represent analog series. The color of the nodes (blue or gray) indicates if a compound is in training (blue) or test sets (gray). For the CPD-Target and AS-Target splitting strategies, training and test compounds can differ across targets. For simplicity, only two targets and shared compounds are represented

ML models were built on the basis of different newly designed data division (splitting) strategies to provide increasingly challenging test systems for the evaluation of ST and MT learning. The different strategies accounted for target-based vs. global data organization as well as compound- or analog series-based splitting and are schematically illustrated in Fig. 1.

#### Per-target vs. global division

Training and tests can be assembled on a per-target basis such that each individual target has its own training and test sets. By contrast, a global splitting strategy assigns each compound either to the global training or test set, regardless of the target. For ST models, training and test sets are always generated in a target-based manner. However, for MT predictions, it must be decided whether a compound should be added to the training or test set.

#### Compound- vs. analog series-based division

Training and test sets can also be generated on the basis of individual compounds or analog series, which consist of compounds sharing the same core structure and having different substituents (R-groups). Data organization based on analog series ensures that compounds with very similar structures will not occur in both training and test sets, which generally increases the difficulty of a prediction tasks compared to compound-based selection. Training and test sets containing distinct analog series challenge prediction models but provide model generalization in successful instances. For our study, all available analog series were algorithmically extracted [21] from our compound collection. In network representations where compound nodes are connected by edges if they are structural analogs, disjoint clusters consist of individual analog series (Fig. 1).

#### Definition of test systems

Applying global or target-based data division as well as compound- or analog series-based division, four different test systems were defined (Fig. 1). First, compounds were randomly divided into training and test sets on per-target basis (CPD-Target). Second, a compound and its activity annotations were exclusively used for training or testing across all targets (CPD-Global). Third, complete analog series were randomly divided into training and test sets on a per-target basis (AS-Target). Fourth, analog series were globally divided (AS-Global).

For simplicity, in Fig. 1, all compounds are annotated against the same two targets. However, in our test systems, a compound might be active against single or multiple targets. The application of global data splitting schemes ensured that a compound (CPD-Global) or an entire analog series (AS-Global) consistently participated in either training or test sets, even if annotations against multiple targets were available.

### General model comparison

ST- and MT-DNN models as well as ST-RFR and ST-SVR models were generated and evaluated using multiple measures including the mean absolute error (MAE), median absolute error (MedAE), mean squared error (MSE), and correlation coefficient (r) values. The mean and standard deviation of these metrics were first calculated for all the models across all targets, as reported in Table 1. The results revealed general trends. Many successful models were obtained, which approached experimental accuracy in a number of instances, with differences between predicted and experimental potency values falling narrowly within an order of magnitude. Moreover, correlation between predicted and experimental potency was overall high, which indicated that well performing models should be applicable to many targets, at least to generate ranking schemes for compound prioritization. As anticipated, other activity classes displayed lower performance levels, which were separately analyzed, as discussed below. The reasonable global model performance across different test systems provided a sound basis for further analysis of the predictive models.

**Table 1:** Global model performance

	ST-DNN	MT-DNN	RFR	SVR
MAE	0.607 ± 0.184	0.568 ± 0.154	0.546 ± 0.146	0.531 ± 0.142
MedAE	0.478 ± 0.184	0.450 ± 0.154	0.436 ± 0.150	0.415 ± 0.140
MSE	0.682 ± 0.475	0.586 ± 0.335	0.536 ± 0.299	0.519 ± 0.291
R	0.656 ± 0.213	0.673 ± 0.198	0.705 ± 0.185	0.714 ± 0.175

Mean (± standard deviation) values are reported for multiple measures (MAE, MedAE, MSE, r) and four types of ML models (ST-DNN, MT-DNN, RFR, SVR)

### Comparison of DNN models

MT-DNN and ST-DNN models were built for all 270 targets. Therefore, a model that predicted compound potency against 270 targets was compared to 270 individual models for single targets. Three independent trials were carried out per method and test system. For these three trials and 270 targets, the percentage of cases with better predictions with MT-DNN or ST models is reported in Table 2. ST models are only trained with compounds having potency values for the particular target. In the absence of annotations for a given target, the compound cannot be added to the training or test sets. In the case of MT-DNN models, all compounds that have at least one potency value for given target can be used. For MT-DNN model training and testing, only available potency annotations were considered in the loss or error calculations. When a compound was not annotated for a given target, the corresponding interaction in the compound-target label matrix was masked in the loss function calculation such that this value was not used for training error estimation. Therefore, only available potency values were used for backpropagation. This masking scheme was also applied to potency values used for model evaluation. Accordingly, test set predictions were exclusively assessed on the basis of compound-target interactions with available K_i_ values.

**Table 2:** Activity classes with superior MT-DNN or ST-DNN performance

Measure	CPD-Target		CPD-Global		AS-Target		AS-Global	
	MT < ST	MT > ST	MT < ST	MT > ST	MT < ST	MT > ST	MT < ST	MT > ST
MAE	4.8	26.0	6.8	13.8	5.3	25.8	10.0	16.9
MedAE	7.5	23.3	12.3	17.5	9.1	26.3	17.9	18.4
MSE	9.5	41.4	14.2	28.0	10.0	42.0	18.3	29.8
r	7.0	19.1	12.0	9.9	8.8	20.1	14.1	14.2

For each data splitting strategy (CPD-Target, CPD-Global, AS-Target, AS-Global) and multiple performance measures (MAE, MedAE, MSE, r), the percentage of compound activity classes having a > 0.1 difference in performance in MT-DNN vs. ST-DNN is reported. “MT < ST” and “MT > ST” refer to better predictions with ST models and MT-DNN, respectively

When comparing MT-DNN and ST model results, the performance was considered superior if errors were at least 0.1 smaller or correlation between predicted and observed potencies was 10% higher. For a confined proportion of activity classes, the performance of MT-DNN model across different test systems was significantly better than of ST-DNN models, as determined using different measures. On the basis of error quantification (MAE, MedAE, MSE), there were more activity classes with accurate predictions for MT-DNN than ST-DNN models. For the basic CPD-Target approach, only 4.8% of the test cases gave at least 0.1 smaller MAE values with ST-DNN compared to MT-DNN, whereas 26% of the cases improved with MT learning. For the same test system, MSE differences were larger, with 9.5% and 41.4% of the activity classes better predicted by ST- and MT-DNN models, respectively. Interestingly, for the most challenging AS-Global test system, MAE calculations revealed comparably small differences. In this case, for 10 and 16.9% of the activity classes, superior performance was observed for ST- and MT-DNN, respectively. On the basis of MSE calculations, the proportions of activity classes with better predictions were 18.3% for ST- vs. 29.8% for MT-DNN models. Overall, there was a consistently detectable advantage of MT- over ST-DNN learning, with a notable influence of the data division strategies on relative model performance. Target-based data division favored MT-DNN learning. Correlation coefficient values were only similar for different ML methods when the per-target splitting strategy was applied, i.e., when test compounds with activity annotations against multiple targets were separately considered for these targets. This provided a generally improved basis for MT- over ST-DNN learning, consistent with our findings.

### Models with different test systems

**Fig. 2:**
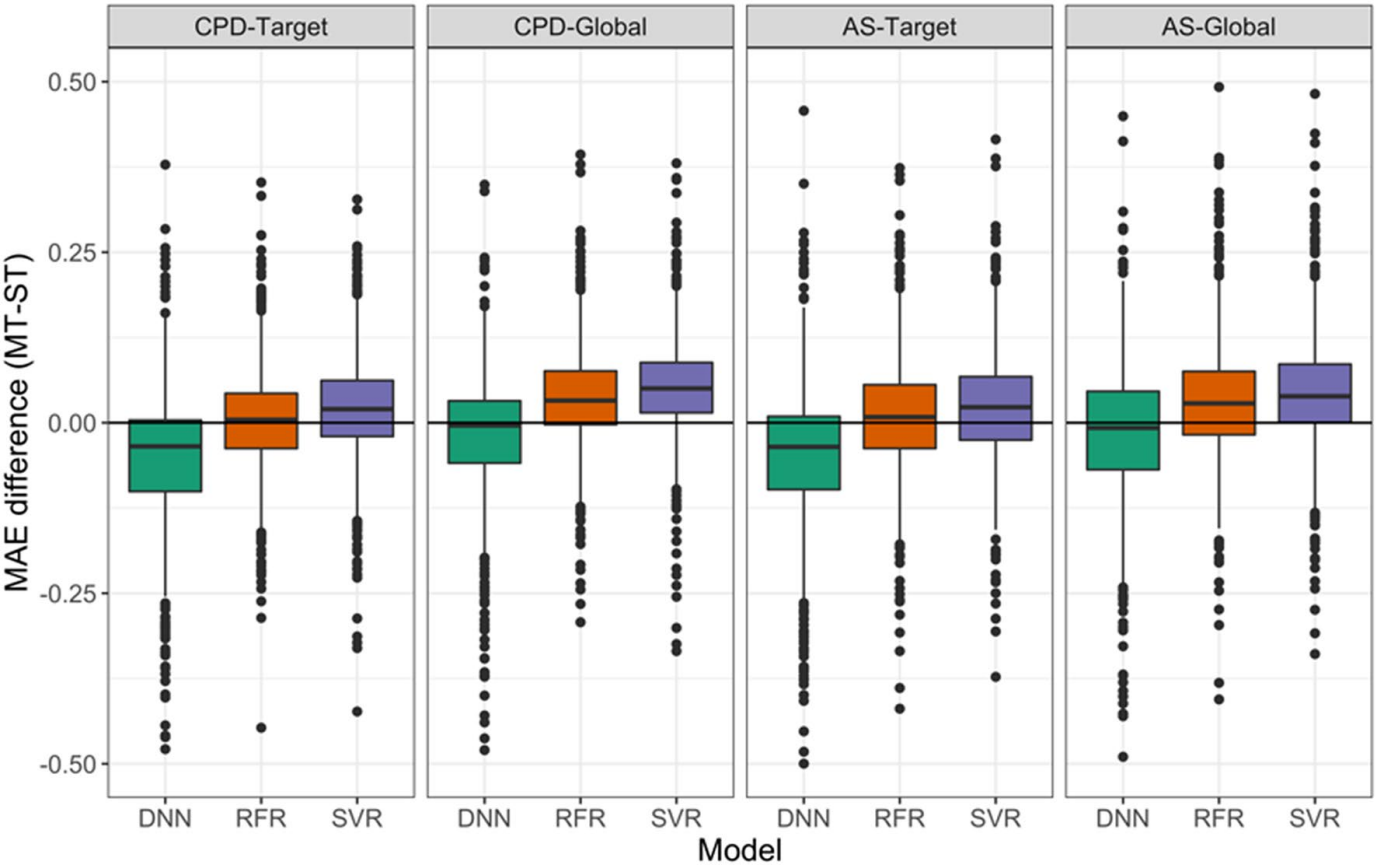
Comparison of MT-DNN and ST models. Boxplots report the distribution of differences in MAE between MT-DNN and ST-DNN, -RFR, and -SVR models for different data division strategies. Negative and positive values indicate larger errors for ST and MT models, respectively. SVR generally provided more accurate predictions, followed by RFR in many instances. DNN distributions are shifted towards lower values for target-based splitting strategies. Boxplots show the smallest value (lower whisker), lower quartile (lower boundary of the box), median (vertical line in box), upper quartile (upper boundary of the box), and the maximum value (upper whisker). Values classified as statistical outliers are represented as black dots

MT-DNN performance has been compared to ST-DNN, RFR, and SVR models. The MAE difference between the MT and ST models was calculated. Figure 2 shows the distributions of MAE differences (MAE_MT_ − MAE_ST_) for all the activity classes and test systems. The distributions contained statistical outliers corresponding to activity classes for which ST or MT models performed substantially better than their counterparts. Overall, SVR models yielded higher performance than other ML models, especially for global test systems. For many targets, potency predictions with SVR- and RFR-ST models were clearly more accurate than those of ST-DNN models. Although there might be specific architectures or hyper-parameter combinations that further boost in performance of DNN models, for hyper-parameter combinations we tested and the increasingly challenging test systems we investigated, there was no advantage over ST-SVR or -RFR models. Importantly, the results also showed that the DNN models were substantially affected by alternative data splitting strategies upon which the different test systems were based. For target-based test systems, ST-DNN models produced larger errors than MT-DNN models, as mentioned above, but for global test systems, median error of these models were nearly identical. Analog series-based data splitting forces models to predict compounds that are dissimilar to the training set, which might be expected to favor DNN models. However, especially for the AS-Global test system, ST-SVR and -RFR models produced lower errors than MT- and ST-DNN models, indicating that DNN model had less potential for generalization.

### Challenging compound sets

In addition to exploring alternative test systems/conditions, large-scale assessment of many different activity classes was another driver of our study. For the 270 classes we investigated, a separation into “suitable” classes, which yielded fairly or highly accurate predictions, and “difficult” classes was observed, for which prediction accuracy was much lower.

**Fig. 3:**
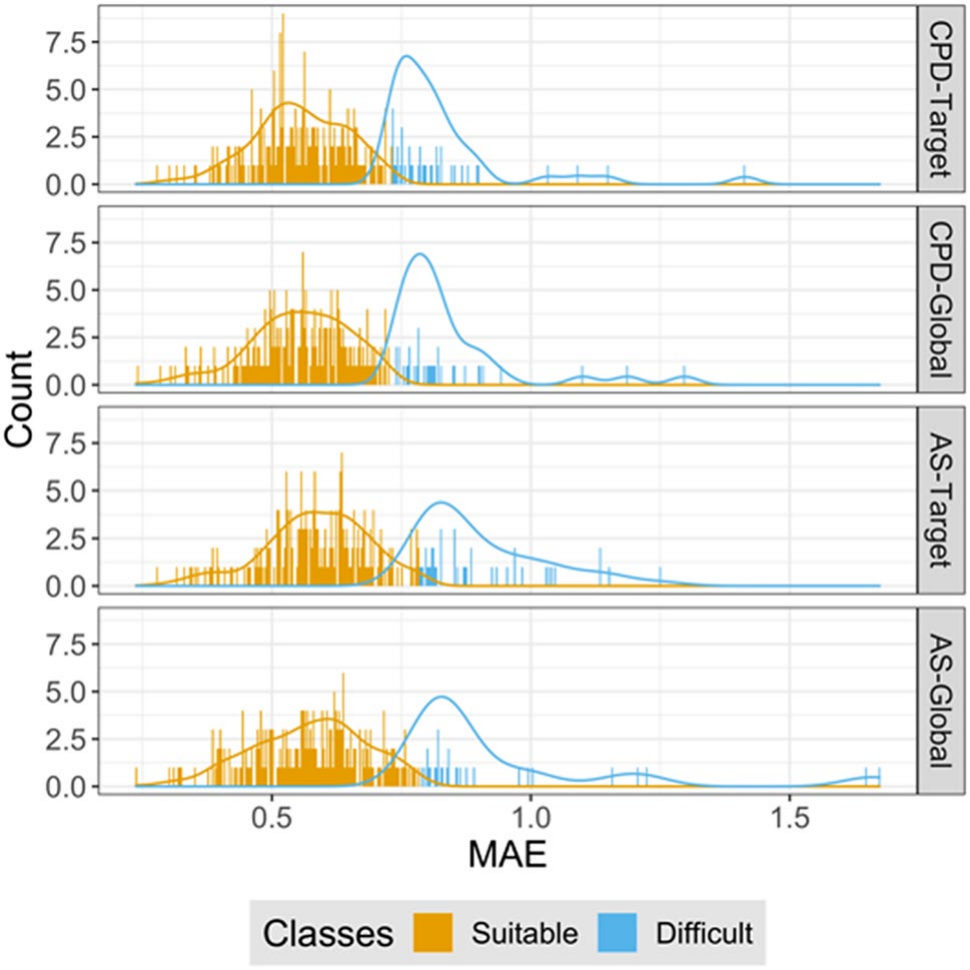
Activity classes with the largest prediction errors. Histograms report the distribution (count) of activity classes over the MAE range for ST-DNN models and different data division strategies. Activity classes with lowest performance (highest prediction error) are colored in blue. These classes represent the most challenging (“difficult”) prediction targets. Activity classes yielding lower prediction errors (“suitable”) are indicated in gold. The threshold criterion distinguishing between suitable and difficult classes was set to the mean error value plus two standard deviations (e ≥ µ + 2σ)

**Fig. 4:**
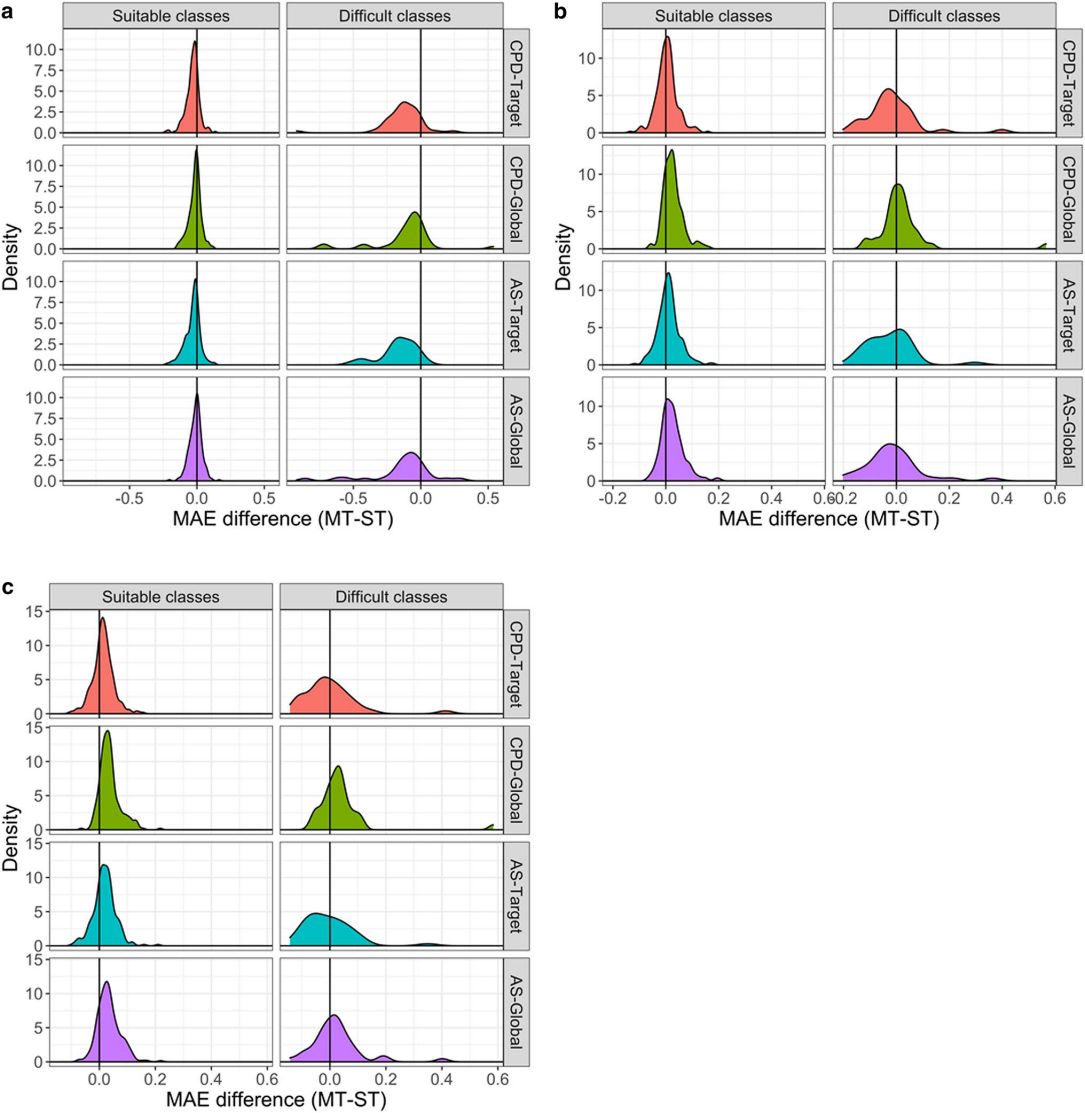
Error difference distribution. For suitable and challenging activity classes according to Fig. 3, density plots show the distributions of MAE differences between MT-DNN and ST models for different data division strategies. **a** ST-DNN, **b** ST-RFR, and **c** ST-SVR

**Fig. 5:**
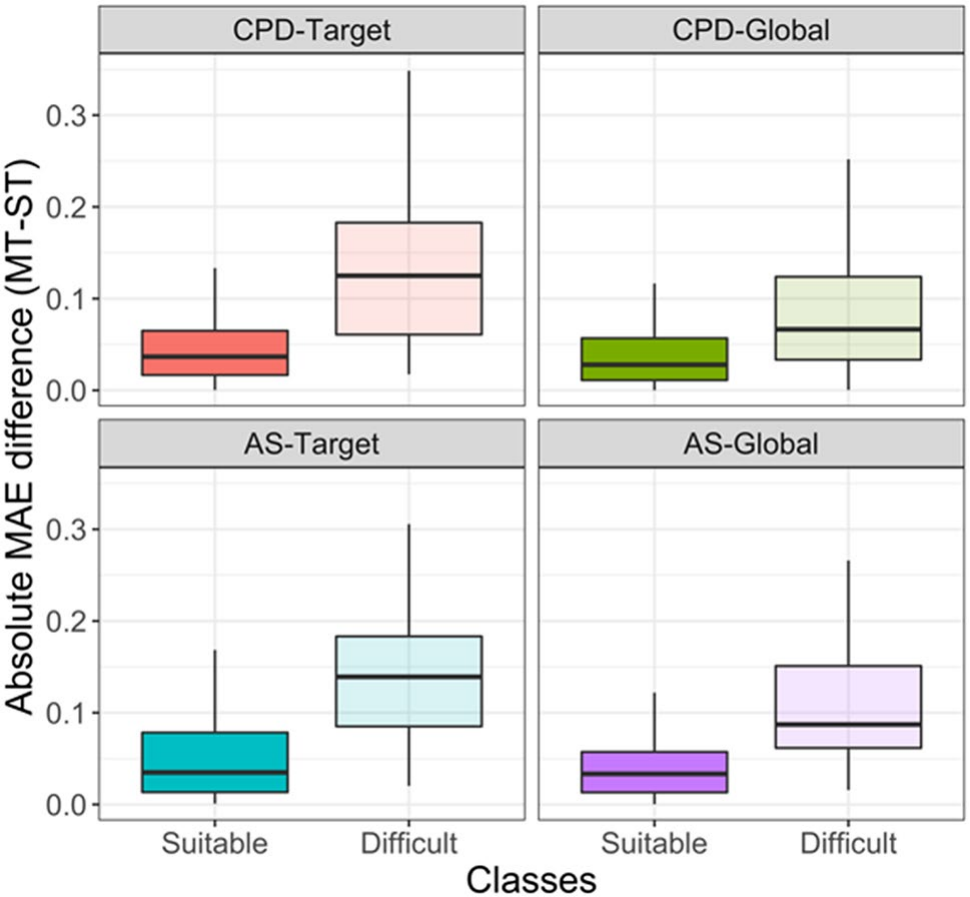
Absolute error differences. Boxplots report the distributions of absolute MAE differences between MT-DNN and ST-DNN models for suitable and challenging activity classes and different data division strategies

To track activity classes that were most challenging for ST models, we set an error threshold criterion to e ≥ µ + 2σ (mean plus two standard deviations) for difficult classes. On the basis of the MAE measure, 40, 34, 40, and 29 activity classes were identified that were difficult for ST-DNN models and the CPD-Target, CPD-Global, AS-Target, and AS-Global test systems, respectively. Figure 3 shows the distribution of activity classes with lowest ST-DNN prediction accuracy compared to the remaining classes. For difficult classes with largest prediction errors, the results were compared to those obtained with MT-DNN models. Figure 4a reports the difference in MAE values between MT-DNN and ST-DNN models. For the most difficult classes, ST-DNN performance was clearly improved using MT-DNN models, as indicated by a one-sided Wilcoxon test applied to the distributions (p values < 0.0001), confirming statistical significance of the observations. Figure 4b, c show the results for corresponding comparisons of and ST-RFR and -SVR models with MT-DNN models, respectively. Statistical tests were also applied in these cases. For RFR/SVR models, the one-sided Wilcoxon test gave p values of 0.003/0.016 (CPD-Target), 0.019/0.099 (CPD-Global), 0.004/0.003 (AS-Target), 0.046/0.199 (AS-Global). Thus, the comparison between distributions for the activity classes with overall lowest performance also revealed an influence of data splitting strategies since smaller p values were obtained for target-based test systems. In particular, differences between MT-DNN and ST-RFR/SVR models were significantly shifted towards negative values for the target-based test systems, indicating a larger performance gain for MT-DNN models. Equivalent results were obtained for statistical significance calculations on the basis of MSE values. For difficult activity classes, performance differences between ST- and MT-DNN models were largest, as further illustrated by the MAE difference distributions shown in Fig. 5.

**Fig. 6:**
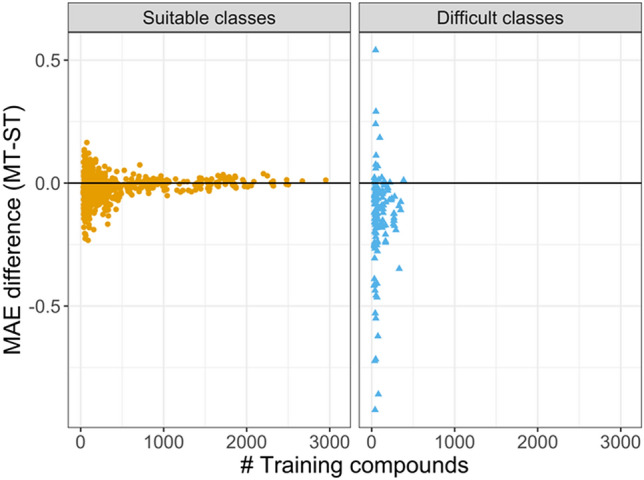
Relationship between training set size and relative model performance. Plotted is the number of training compounds vs. the difference between MT and ST model performance for suitable and challenging activity classes

For difficult activity classes with statistically significant improvements in prediction accuracy via MT-DNNs, the number of available training set instances was examined. Figure 6 shows the difference in the number of training instances available for MT-DNN and ST-DNN models. For suitable classes, increasingly large numbers of training compounds were available in many cases, which led to balanced performance of ST- and MT-DNN models. For small numbers of training compounds, moderate performance variations were observed. By contrast, for difficult classes, only small numbers of training instances were generally available, but large-magnitude differences in prediction errors were observed, which could not entirely be attributed to the limited training data, as the comparison with suitable activity classes showed. Nonetheless, small training sets were a characteristic of difficult classes. For ST-DNN models, the median number of training set compounds was 64 for difficult and 134 for suitable activity classes.

### Control calculations

**Fig. 7:**
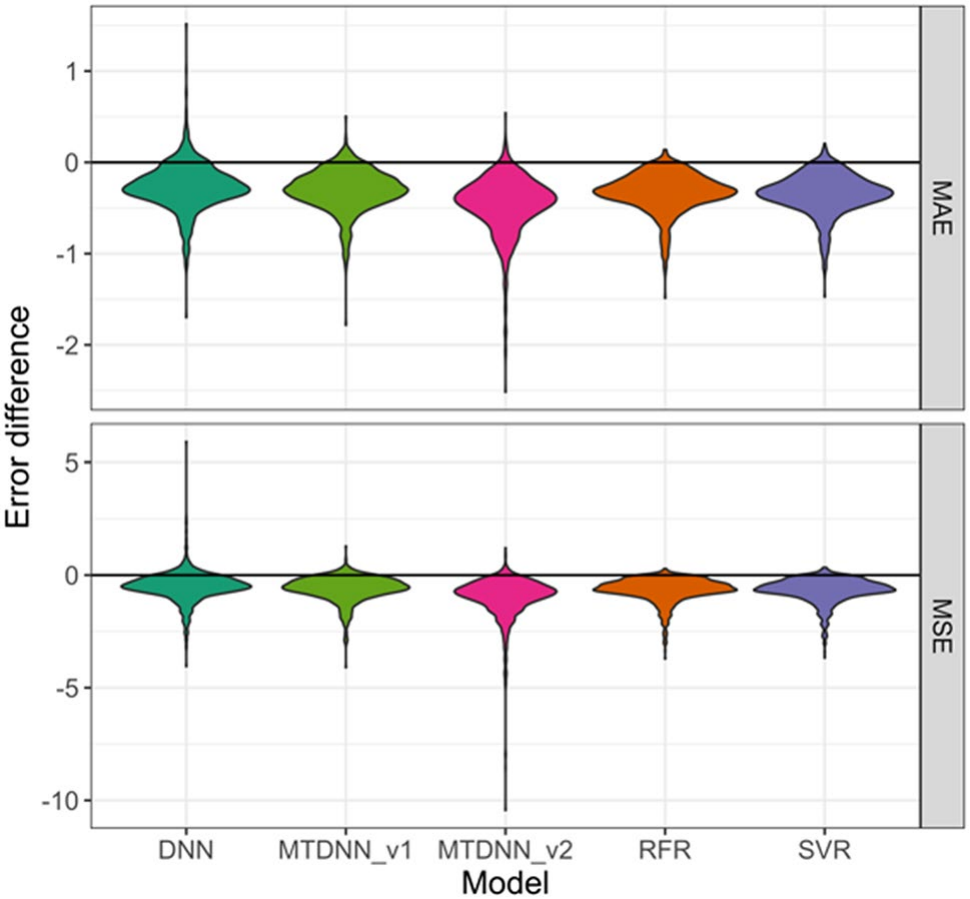
Reference state for predictions. For different types of models, violin plots report the distributions of MAE and MSE values for test set potency predictions relative to differences to the calculated mean potency of the training set. Positive values indicate that predicting the calculated mean pK_i_ value yields better results than the actual model. MT-DNN_v1 and v2 refer to comparisons to the average over individual tasks or the average across all tasks, respectively. Replacing predictions with mean training set potency consistently led to larger errors compared to MT or ST models

Considering the presence of dynamic data ranges for learning, further control calculations were carried out by comparing predictions to the mean potency of training set compounds. Use of the mean potency as an approximation would not require any learning. Figure 7 shows that replacing predicted potency values with the mean training set potency led to increasing errors. Only a small proportion of the regression models did not benefit from learning. In total, there were 1080 model instances, corresponding to combinations of 270 activity classes and four test systems. Table 3 reports the number and percentage of cases with equivalent or better results when predicted values were replaced with mean training set potencies. With 13.6%, the proportion of such models was by far largest for ST-DNNs. MT-DNN model errors were compared to assigning the mean training set potency of a particular compound class or the average across all classes. Since different activity classes had distinct dynamic data ranges, MT-DNN target-based model predictions were generally more accurate than using the global mean as a control.

**Table 3:** Regression models with most limited training success

Models	Prediction instances
ST-RFR	2.4% (26)
ST-SVM	3.6% (39)
ST-DNN	13.6% (146)
MT-DNN_v1	7.3% (79)
MT-DNN_v2	3.3% (35)

For model category, the percentage (number) of models with prediction errors comparable to those obtained when replacing predicted vales with mean training set potency is reported. MT-DNN_v1 and v2 refer to comparisons to the average over individual tasks or the average across all tasks, respectively

## Conclusions

In this work, we have investigated the influence of alternative data division strategies on different ST models and MT-DNN models and carried out large-scale potency value predictions over many qualifying activity classes. For these purposes, increasingly challenging test systems were defined for model training and evaluation. Overall, promising predictions were obtained that approached experimental accuracy in some instances. Furthermore, there was only little advantage of DL over other ML models. However, the analysis revealed that alternative data splitting strategies and the resulting test systems influenced the relative performance of models in different ways. Difficult activity classes with large prediction errors were identified for which typically only limited training data were available. However, low prediction accuracy could only partly be attributed to data limitations. When MT-DNN models were evaluated on compounds that were included in the training sets for other targets, the obtained results were generally superior to ST models. In cases where ST model accuracy was limited, largest relative performance gains of MT-DNN models were observed. In light of our findings, model training can be adjusted to different conditions, depending on specific application tasks. For example, when data sets with training and test sets comprising distinct analog series are used, generalization potential of ST and MT models is often the highest.
